# Effects of Harvest Time and Hydrodistillation Time on Yield, Composition, and Antioxidant Activity of Mint Essential Oil

**DOI:** 10.3390/molecules28227583

**Published:** 2023-11-14

**Authors:** Samara de Paula Pinheiro Menezes Marques, Rafaela Oliveira Pinheiro, Rafael Alves do Nascimento, Eloísa Helena de Aguiar Andrade, Lênio José Guerreiro de Faria

**Affiliations:** 1 Programa de Pós-Graduação em Engenharia dos Recursos Naturais da Amazônia, Universidade Federal do Pará, Belém 66075-110, Pará, Brazil; samara@ufpa.br; 2 Faculdade de Engenharia Química, Universidade Federal do Pará, Belém 66075-110, Pará, Brazil; rafaelap@ufpa.br; 3 Laboratorio Adolpho Ducke, Coordenação de Botânica, Museu Paraense Emílio Goeldi, Belém 66077-830, Pará, Brazil; eloisa@museu-goeldi.br

**Keywords:** *Mentha spicata* L., menthol, gas chromatography, diurnal variation

## Abstract

In this study, we assessed the effects of different harvest times (9 a.m., 1 p.m., and 5 p.m.) and hydrodistillation times (60, 90, and 120 min) on the yield, chemical composition, and antioxidant activity of the spearmint (*Mentha spicata* L.) essential oil (EO) sourced from the Amazon region. EO yield was ≥1.55% and was not significantly influenced (*p* ≥ 0.05) by the different harvest times and hydrodistillation times. Thirty-one different organic compounds were identified, of which menthol (91.56–95.68%), menthone (0.6–2.72%), and isomenthone (0.55–1.46%) were the major constituents. The highest menthol content in the EO was obtained from samples collected at 9 a.m., with a hydrodistillation time of 60–90 min, compared to other harvest and hydrodistillation times. This suggests that exposure to sun and light, which is greater at harvest times of 1 p.m. and 5 p.m., decreased the menthol content and altered the chemical composition of Mentha EO. Furthermore, the sample harvested at 9 a.m. and hydrodistilled for 60 min showed the highest antioxidant activity (61.67 equivalent mg of Trolox per g of EO), indicating that antioxidant activity is strongly affected by light exposure and the contact duration of the sample with boiling water during hydrodistillation.

## 1. Introduction

*Mentha spicata* L., popularly known as mint or mentha, is a plant that belongs to the genus mentha or menthe, comprises 25 to 30 species, is cultivated on five continents, and has commercial relevance [1]. These species exhibit favorable pharmacological properties and are applicable in the food industry. Moreover, mint oils are among the most important essential oils (EO) produced in the world, and their values exceed USD 400 million per year [2].

EOs from *Mentha spicata* L. are widely used in the food, cosmetics, perfume, beverage, pharmaceutical, and tobacco industries [3]. Ćavar Zeljković et al. [4] reported that mint is rich in phenolic compounds, which exhibit antioxidant properties that are useful in the food industry. The EOs from *Mentha spicata* L. have been incorporated in several products, such as toothpastes, mouthwashes, perfumes, and beverages [5].

EOs such as mint oils are generally complex mixtures of hydrocarbon monoterpenes and sesquiterpenes, oxygenated monoterpenes and sesquiterpenes, and other compounds derived from the secondary metabolism in plants [6], and they are increasing in the pharmaceutical, food, chemical, and cosmetic industries [7]. However, a reliable and standard production method for the desired quantity and quality of EOs has not been reported thus far. Therefore, studies on the harvesting and processing conditions of *Mentha spicata* L. are necessary to overcome such deficiencies.

Environmental factors such as harvest time (HT) and other technical-industrial factors such as hydrodistillation time (HDT) influence the EO yields. El Kharraf et al. [8] suggested that the method, extraction device, and extraction time of EO production should be customized to each species, aiming to obtain the highest yields.

Harvest time is related to both the environment and the physiology of the plant. Oliveira et al. [9] reported that two simultaneous response patterns exist in the secondary metabolism of plants to environmental stimuli: the larger and slower-paced seasonal climatic variations and the smaller and faster daily climatic fluctuations. Rguez et al. [10] indicated that harvest time is an important parameter for EO production as the chemical composition of plants continuously changes throughout the day.

According to Sintim et al. [11], the hydrodistillation time can affect the yield, bioactivity, and composition of essential oils. Thus, HDT can be used to obtain oils with desired compositions and bioactive activities that can be used for specific therapeutic purposes. Furthermore, HDT is a good process parameter and can be used to predict operational costs [12].

Mint oil has nutraceutical benefits and high economic value. Thus, several studies focused on optimizing its yield and determining the factors that influence its quality and quantity during extraction [13,14]. However, the factors that affect the yield and quality of mint EO grown in the Amazon have not been investigated to date. Hence, in this study, we investigated the influence of HT and HDT on the yield, chemical composition, and antioxidant activity of mint EO.

## 2. Results and Discussion

### 2.1. Effect of HT and HDT on EO Yield

HT and HDT showed no significant effects on EO yield (*p* > 0.05), as shown in Table 1. Tukey’s test revealed no significant differences between the average yields for different HTs (9 a.m., 1 p.m., and 5 p.m.) and HDTs (60 min, 90 min, and 120 min).

Bufalo et al. [15] analyzed the effects of harvest times on the production of EO from *Mentha spicata* L. from the United States and reported significant yield variations in samples harvested at 1 p.m. and 9 a.m. This difference between the results of Bufalo et al. [15] and those shown in Table 1 (in which the different harvest times did not influence the EO yield) could be attributed to different climatic conditions such as temperature, light, relative humidity, and soil nutrients in the harvest location, as well as genetic factors intrinsic to the species that are affected by environmental factors.

In addition, Figueiredo et al. [16] suggested that different plants are affected differently by environmental factors, which in turn affects EO yield. Plants have different behaviors during diurnal variation. Some species accumulate more oil [17], and some produce less oil [18]. *Mentha spicata* L. produces a reliable quantity of oil when harvested between 9 a.m. and 5 p.m.

However, thus far, no study has investigated the influence of HDT on the EO yield from *Mentha spicata.* L. Oliveira et al. [9] observed a stable oleic acid yield from *Mentha x piperita* var *citrate* after 60 min of hydrodistillation in a Clevenger device; however, the yield did not change at the HDT of 90 or 120 min. Furthermore, Zheljazkov, Astatkie, and Schlegel [15] reported stable EO yields from *coriander* (*Coriandrum sativum*) between 40 and 160 min of hydrodistillation, following which the yield declined significantly at an HDT of 240 min.

Researchers attributed this optimal HDT to the diffusion process in the internal parts of the plant. After the first phase of rapid extraction of EO from the external surface of the plant, the slower process of internal diffusion involving physical modifications to the cells containing EO promotes the extraction of the oil in the presence of water vapor [19]. Therefore, in the case of *Mentha spicata* L., the HDTs of 60, 90, and 120 min were sufficient to achieve internal diffusion and are optimal EO extraction times.

**Figure 1:**
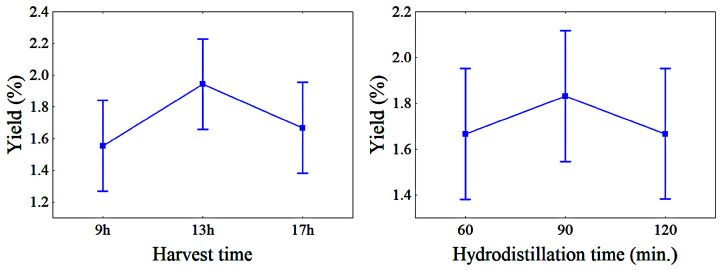
Variation in the EO yield with HT and HDT; bars denote a 95% confidence interval.

The mean EO yields as a function of HT and HDT are shown in Figure 1. The values ranged from 1.55% to 1.94% for different HTs and from 1.66% to 1.83% for different HDTs.

The mint EO yields extracted by hydrodistillation in this study are consistent with those reported by Narasimhamoorthy et al. [20], Giatropoulos et al. [21], and Kripanand, Guruguntla, and Korra [22] extracted from the United States (1.73%), Greece (1.60%), and Iran (1.89%), respectively. Moreover, our yields are higher than those obtained by Kedia et al. [23] and Sousa Barros et al. [24], who harvested from India (0.7%) and Brazil (0.17%), respectively. According to the criteria proposed by Correa Junior, Ming, and Scheffer [25], our EO yields are excellent and promising for commercial production as the values are higher than 1%.

The selection of ideal conditions for the production of mint EO should consider not only the quantitative yield but also the cost of production. Ideally, mint EO production should require the shortest time possible while maintaining a high yield. Hence, HDT should require low energy consumption, leading to lower production costs. Under the operating conditions used in our study, an HT between 9 a.m. and 5 p.m. is ideal, which prevents loss of yield of the mint EO during production.

### 2.2. Effect of HT and HDT on the Chemical Composition of EO

The chemical composition of the EOs extracted from *Mentha spicata* L. is shown in Table 2. Thirty-one components have been identified, and their amounts varied with different HTs and HDTs.

The samples collected at 5 p.m. and 1 p.m. have 24 components, whereas those collected at 9 a.m. have 22 constituents, thus showing that the duration of exposure to solar radiation leads to higher component variability. Oxygenated monoterpenes (97.79–99.40%) were the highest class of compounds, followed by hydrocarbon sesquiterpenes (0.62–1.53%), hydrocarbon monoterpenes (0.05–0.71%), and oxygenated sesquiterpenes (0.05–0.12%). Furthermore, the major components were menthol (91.56–95.68%), menthone (0.6–2.72%), and isomenthone (0.55–1.46%).

The results also indicate qualitative variations in the EOs. Cis-3-hexenyl isovalerate was absent only in the 9 a.m. EOs, whereas isopulegol and (*Z*)-3-hexenyl-2-methylbutyrate were detected only in the 9 a.m. EOs. Moreover, hydrocarbon monoterpenes such as α-pinene, sabinene, and β-pinene had higher concentrations or were uniquely identified in the samples collected at 1 p.m. and 5 p.m. Marchese and Figueira [26] attributed this phenomenon to the photosynthesis-dependent biosynthesis of hydrocarbon monoterpenes.

Furthermore, the amounts of some EO components depend on temporal climatic changes during the day [27]. According to Gobbo-Neto and Lopes [28], daily temperature fluctuations and UV radiation levels are among the factors influencing secondary metabolite production. Although variations in ambient temperature and UV radiation were not closely monitored in our study, it is worth noting that in the Amazon region, temperatures are lower at 9 a.m. and UV radiation is less intense than at 1 p.m. and 5 p.m. This may have influenced the biosynthetic pathway of production of monoterpene hydrocarbons, as these compounds are typically synthesized when the plant is exposed to higher temperatures and greater rates of UV radiation [27,28].

The HDT had a significant effect (*p* < 0.05) on the menthol and menthone content (the main components of the EO), as did the HT, as shown in Table 3 and Table 4, respectively.

The results of Tukey’s test indicated a significant difference between the mean menthol and menthone values at different HCs and HTs (Table 3 and Table 4), showing that the mint harvested at 9 a.m. and with a hydrodistillation time of 60 min. had a higher menthol content in its EO than that from samples with different HTs. However, for the menthone concentration, the opposite behavior was observed: the highest content of this constituent was quantified in the sample collected at 5 p.m. and with a HDT of 120 min.

**Figure 2:**
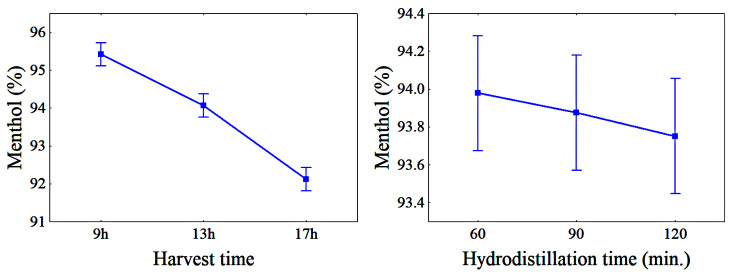
Variation of the menthol content with HT and HDT; bars denote a 95% confidence interval.

**Figure 3:**
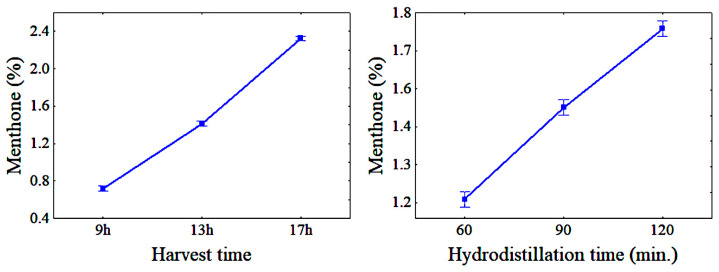
Variation of the menthone content with HT and HDT; bars denote a 95% confidence interval.

This inverse correlation can be seen in Figure 2 and Figure 3 and may be related to the menthol oxidation process, which, as hydrodistillation progresses, tends to produce menthone due to the attack of the oxidizing agent in the medium on the secondary carbon linked to the hydroxyl present in the menthol structure. In addition, according to Figueiredo et al. [16], the longer the hydrodistillation takes, the greater the possibility of variation in the chemical composition of the EO due to the chemical transformations that the plant matrix undergoes because of the effect of the heat.

The influence of harvest time on the production of menthol and menthone in Mentha EOs has already been reported in the literature. According to Shiwakoti et al. [29], a higher rate of light radiation tends to oxidize the chemical constituents of EO. The highest menthol content was observed in samples collected at 9 a.m., followed by 1 p.m. and 5 p.m. On the other hand, the highest menthone content was quantified in samples collected at 5 p.m., followed by 1 p.m. and 9 a.m. Based on these results, it can be inferred that greater solar radiation affects the activity of the limonene 3-hydroxylase enzyme, thereby decelerating the natural process of converting menthone into menthol, as described by Croteau [30].

Figure 2 and Figure 3 show, respectively, the variation of the mean menthol and menthone content concentration in the EO from *Mentha spicata* L. with different HTs and HDTs. The menthol content ranged from 92.12% to 95.43% and menthone from 0.72% to 2.33% for different HTs. For different HDTs, the menthol content ranged from 93.75% to 93.98% and menthone from 1.21% to 1.75%.

Kowalczyk et al. [31] and Telci et al. [32] reported that menthol content in the EO from aromatic plants changes with HT and attributed it to factors such as geographical origin. In addition, the duration of light and sun exposure affects the phytochemistry of the plant. Samples harvested in the morning show the accumulation of certain components that are necessary to respond to the environmental changes that occur during the rest of the day [33].

However, a consensus has not been reached on the chemical composition and major components of *Mentha spicata* L. to date. The major components in the samples grown in India [23] are dextro-carvone (59.6%), limonene (25.59%), and m-cymene (2.77%); those from the United States [34] are carvone (70.36%), limonene (6.6%), and β-myrcene (2.41%); those from Egypt [35] are menthone (32.43%), 1,8-cineole (18.79%), and cis-isopulegone (16.65%); those from Tunisia [36] are l-menthone (32.74%), pulegone (26.67%), and menthol (11.42%); and those from Italy [37] are carvone (62.9%), limonene (8.5%), and 1,8-cineole (6.0%). These reports are not consistent with the major components identified in this study: menthol, menthone, and isomenthone. This suggests that the species have more than one chemotype.

This inference is supported by a previous report by Baser et al. [38], who reported three *Mentha spicata* L. chemotypes in Turkey. They reported that menthone/isomenthone, trans-sabinene/carvone/terpinen-4-ol hydrate, and 1,8-cineole/linalool/carvone are the major components in *Mentha spicata* L. Other phytochemical patterns of *Mentha spicata* L. EO have also been identified in Brazil (Table 5). However, the sample cultivated in the Amazon in this study has the highest menthol content.

Menthol has applications in the chemical, pharmaceutical, and cosmetic industries. Several studies reported its anti-inflammatory, antifungal, antiviral, immunomodulatory, antitumor, analgesic, and neuroprotective activities [43,44,45,46,47,48]. Moreover, menthol accounts for approximately USD 400 million annually, with a gross production of ~7000 metric tons [2,30]. This large market demand necessitates new raw materials and improved production processes for menthol.

### 2.3. Effect of HT and HDT on the Total Antioxidant Activity (TAA) of the EO

HT and HDT significantly affected the TAA of EO, expressed as I (%) (*p* ≤ 0.05), as shown in Table 5. Tukey’s test also revealed significant differences between the mean percentage inhibition values of 2,2-diphenyl-1-picrylhydrazyl (DPPH) radicals for different HTs and HDTs (Table 6).

**Figure 4:**
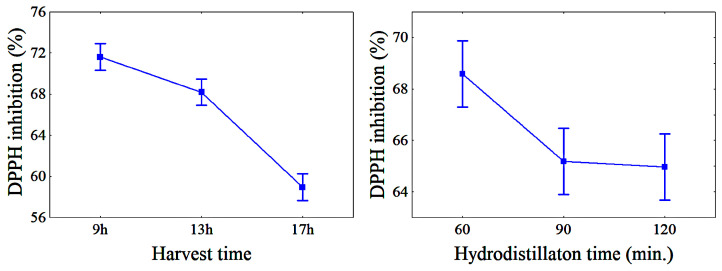
Variation of TAA with HT and HDT; bars denote a 95% confidence interval. Abbreviations: DPPH—2,2-diphenyl-1-picrylhydrazyl radical.

Figure 4 shows the mean TAA values [I (%)] as a function of HTs and HDTs. I (%) decreased as HT increased. The mean TAA values for 9, 13, and 17 h of HT were 71.61%, 68.19%, and 58.94%, respectively, indicating that harvesting the material at 9 h is preferable over that at 13 and 17 h.

This behavior could be attributed to the lower light exposure of the samples at 9 am. Trevisan et al. [49] reported that antioxidant activity is strongly affected by light exposure. Plants cannot counterbalance the production of antioxidant components under oxidative stress promoted by high luminosity rates. Rguez et al. [10] also reported that the *Salvia officinalis* EO samples collected at 7 a.m. exhibited a higher TAA than those collected at 12 and 5 pm. Furthermore, the behavior of antioxidant activity described in Figure 4 is also associated with the greater presence of menthol content in the EO of samples collected at 9 a.m. (see Figure 2). According to Nascimento et al. [33], samples harvested in the morning show the accumulation of certain components that are necessary to respond to the environmental changes that occur during the rest of the day.

The EO of *Mentha spicata* L. showed a high DPPH inhibition (68.52%) at a HDT of 60 min (Figure 4), following which the TAA decreased for an HDT of 90 min (65.18%) and stabilized at an HDT of 120 min (64.97%) (Table 6 and Figure 4).

Our results are consistent with those of Sintim et al. [11], who observed that a low HDT for the EO of dill seeds (*Anethum graveolens*) resulted in high antioxidant activity. Therefore, the TAA of an EO depends on the duration for which the sample is in contact with boiling water. High HDTs degrade the thermosensitive components in plant materials [50].

The antioxidant capacity, expressed as the Trolox (6-hydroxy-2,5,7,8-tetramethylchroman-2-carboxylic acid) equivalent (TE), of the *Mentha spicata* L. EO sample collected at 9 a.m. with an HDT of 60 min was 61.67 mg TE g^−1^. This indicates that 1 g of EO is equivalent to 61.67 mg of Trolox, an antioxidant widely used in biological and biochemical applications.

The antioxidant capacity of the *Mentha spicata* L. EO was higher than that observed for other EOs from different raw materials (Table 7). This result is significant because *Mentha spicata* L. is widely available in the Amazon region and easy to cultivate and adapt, which facilitates its sustainable management.

Moreover, the high antioxidant character of *Mentha spicata* L. EO could be explained by the major components menthol and menthone, which combat free radicals [14,51].

**Table 7:** Comparison of the TAA (mg TE g^−1^ EO) of *Mentha spicata* L. EO with those of other EOs obtained from different raw materials.

Essential Oils	TAA (mg TE g^−1^ EO)	Reference
*Mentha spicata* L.	61.67	This study
*Eugenia uniflora* L.	186.9	Costa et al. [52]
*Calendula officinalis* L.	2.94	Ak et al. [53]
*Pinus halepensis* Mill.	0.31	Khouja et al. [54]
*Lippia alba* (Mill.) N.E.Br. ex Britton & P. Wilson	51.9	Barros et al. [55]
*Mentha piperita* L.	19.79	Pavlić et al. [56]
*Cannabis sativa* L., var. “Kompolti”.	8.63	Palmieri et al. [57]

## 3. Materials and Methods

### 3.1. Materials

Mint leaves and branches [*Mentha spicata* L.; specimens are stored in the herbarium of the Brazilian Agricultural Research Corporation (EMBRAPA), Pará, Brazil (registration ID: IAN201468)] were used as the raw material. The samples were collected in the city of Ananindeua, Pará State, Brazil (coordinates 1°24′34.5″ S 48°23′52.7″ W) in the month of March (Amazon winter) at 9 a.m., 1 p.m., and 5 p.m. using previously sanitized materials. After collection, the samples were properly stored until further use. The moisture content of the samples was determined by the Dean-Stark analytical procedure using dichloromethane [58] as the solvent.

### 3.2. Hydrodistillation and Yield

Approximately 30 g of the material was used for each experimental assay. The samples underwent a hydrodistillation process for 60, 90, and 120 min in a modified Clevenger-type glass extractor attached to a refrigeration system to maintain the water at ~13 °C. The essential oil yield was expressed as an oil percentage in relation to the leaf dry matter content.

### 3.3. Chemical Composition

A 2 µL aliquot of the sample was dissolved in 0.5 mL of 99% UV/HPLC spectroscopic grade hexane (Sigma-Aldrich, San Luis, MI, USA) for analysis. Samples were analyzed on a GCMS-QP2010 Ultra system (Shimadzu Corporation, Tokyo, Japan) equipped with an AOC-20i autoinjector and a GCMS-Solution system containing the Adams [59] and FFNSC 2 (Mondello, 2011) library systems. A DB-5ms silica capillary column (30 m × 0.25 mm; 0.25 μm thick) (Restek Corporation, Bellefonte, PA, USA) was used.

The analysis conditions were as follows: injector temperature, 250 °C; oven temperature programming, 60–250 °C (3 °C min^−1^); carrier gas, He at a linear speed of 36.5 cm s^−1^; injection without flow splitting; electron impact ionization, 70 eV; temperature of the ionization source and connecting parts, 220 °C [60]. Quantification of the volatile components was obtained by peak area normalization using a GC 2010 Plus Series coupled to a FID detector operated under conditions similar to those used for GC-MS except for the carrier gas, which in this case was H_2_. The identification of the chemical components was based on the linear retention index (RI) and previously reported MS fragmentation patterns [59].

### 3.4. Antioxidant Activity

The antioxidant activity of EO was assessed using the DPPH inhibition method. A stock solution of DPPH (0.5 mM) was prepared using ethanol, and Tween 20 (0.5%, *m*/*m*) was used as an emulsifier for the oil-water mixture. Each EO sample (10 μL) was mixed with 900 μL of Tris-HCl buffer (100 mM, pH 7.4), 40 μL of ethanol, 50 μL of Tween 20 solution, and 1 mL of DPPH stock solution. The control sample was prepared under the same conditions, replacing the EO sample with ethanol. The mixtures were kept in a dark environment at a temperature of ~25 °C. Absorbances were acquired on a UV-1800 (Shimadzu, Kyoto, Japan) UV-Vis spectrophotometer at 517 nm. Readings were recorded at the beginning of the reaction (time zero), every 5 min for the first 20 min, and in continuous 30 min intervals until constant absorbance.

The calibration curve was prepared using Trolox (Sigma-Aldrich, San Luis, MI, USA) at 160–1000 μM. DPPH radical inhibition was expressed as a percentage (%) and TAA was expressed as equivalent milligrams of Trolox per gram of EO (mg TE g^−1^), as described by Choi et al. [61]. All experiments were performed in triplicate.

### 3.5. Statistical Analysis

The yields, chemical compositions, and antioxidant activities were analyzed by ANOVA in randomized blocks, and their means were compared using Tukey’s test at a significance level of α = 0.05. To validate these conclusions, tests for homogeneity of variances (Bartlett and Levene test) and normality of variance (Kolmogorov–Smirnov and Anderson–Darling) were performed with the experimental data at α = 0.05.

## 4. Conclusions

The yield of the essential oil extracted from *Mentha Spicata* L. was ≥1.55% and not statistically influenced (*p* ≥ 0.05) by the proposed changes of harvest times (9 a.m., 1 p.m., and 5 p.m.) and hydrodistillation time (60, 90, and 120 min). Thus, a harvest window of 9 a.m. to 5 p.m. and the shortest hydrodistillation time (60 min) would afford the EO without yield loss. These results could promote the scale-up and continuous production of mint EO.

Thirty-one different organic compounds were identified in the mint EO. The major components were menthol (91.56–95.68%), menthone (0.6–2.72%), and isomenthone (0.55–1.46%). The highest menthol concentration was obtained when the harvest time was 9 a.m., and no significant change (*p* ≥ 0.05) in the results was observed until 90 min of hydrodistillation. These results indicate that the duration of light and sun exposure significantly affects the phytochemistry of the corresponding plant and directly impacts the production of its components.

An inverse correlation between menthol and menthone was observed and may be related to the menthol oxidation process during hydrodistillation. It also might be related to a possible peculiar characteristic of the plant in accumulating, mainly in the morning, components that are more capable of responding to environmental changes during the rest of the day, such as chemical components that combat oxidative-reductive processes. This is the reason why the EO obtained from the harvest performed at 9 a.m. with a hydrodistillation time of 60 min showed the highest TAA (61.67 mg TE g^−1^), indicating that the TAA is strongly affected by menthol concentration, light exposure, and the contact time with boiling water during hydrodistillation.

Therefore, based on the operating conditions used in this study, the best harvest time for the raw material is 9 a.m., and increasing the hydrodistillation time above 60 min does not offer an industrial or economic advantage. Under this condition, it is possible to obtain a mint EO with a good yield, good antioxidant properties, and a low operating cost.

## Figures and Tables

**Table 1:** Analysis of variance (ANOVA) and Tukey’s test for EO yield.

ANOVA					Tukey’s Test				
Effects	df	SS	MS	*p* *	HT **	HDT **			
						60 min	90 min	120 min	Average HT ***
HT	2	0.719	0.360	0.906	9 h	1.499 ^a^	1.498 ^a^	1.668 ^a^	1.555 ^A^
HDT	2	0.164	0.082	0.978	13 h	1.833 ^a^	2.165 ^a^	1.833 ^a^	1.944 ^A^
Error	23	83.359	3.624		17 h	1.668 ^a^	1.835 ^a^	1.501 ^a^	1.668 ^A^
Total	27	84.242			Average HDT ***	1.667 ^A^	1.833 ^A^	1.667 ^A^	

Abbreviations: df—degree of freedom; SS—sum of squares; MS—mean of squares; p—probability of significance (* significant if *p* ≤ 0.05); HT—harvest time; HDT—hydrodistillation time; ** mean values followed by equal lowercase letters in the row and column are not differentiated at 5% probability; *** mean values followed by equal upper case letters are not differentiated at 5% probability.

**Table 2:** Chemical composition of mint EO for different HTs and HDTs.

RIc	Components	9 h			13 h			17 h		
		60 min	90 min	120 min	60 min	90 min	120 min	60 min	90 min	120 min
		Relative Concentration (%) *								
928	α-pinene	-	-	0.04 ^a^	0.06 ^b^	0.05 ^a,b^	-	0.06 ^b^	0.09 ^c^	0.08 ^c^
963	Sabinene	-	-	-	0.05 ^a^	-	-	0.04 ^a^	0.05 ^a^	0.05 ^a^
966	β-pinene	-	-	0.07 ^a^	0.1 ^b^	0.05 ^c^	-	0.07 ^a^	0.1 ^b^	0.08 ^a^
982	3-octanol	0.19 ^a^	0.36 ^b^	0.38 ^b^	0.43 ^c^	0.36 ^b^	0.34 ^b,d^	0.30 ^d^	0.54 ^e^	0.35 ^b^
1018	Limonene	-	0.05 ^a^	0.13 ^b^	0.33 ^c^	0.22 ^d^	0.1 ^b^	0.2 ^d^	0.47 ^e^	0.35 ^c^
1094	Linalool	0.2 ^a^	0.2 ^a^	0.24 ^b^	0.06 ^c^	-	-	-	-	-
1143	Isopulegol	0.13 ^a^	0.51 ^b^	0.12 ^c^	-	-	-	-	-	-
1153	Menthone	0.72 ^a^	0.6 ^a^	0.84 ^b^	1.11 ^c^	1.44 ^d^	1.69 ^e^	1.80 ^e^	2.46 ^f^	2.72 ^g^
1165	Isomenthone	1.44 ^a,e^	0.55 ^b^	0.9 ^c^	1.35 ^d^	1.36 ^d,e^	-	1.46 ^a^	1.40 ^a,d,e^	1.04 ^f^
1166	Neomenthol	-	-	-	-	-	1.65 ^a^	-	-	-
1196	Menthol	95.68 ^a^	95.28 ^b^	95.30 ^a,b^	93.47 ^d^	94.33 ^e^	94.39 ^e^	92.79 ^f^	92.02 ^g^	91.56 ^h^
1197	Neoisomenthol	-	-	-	-	-	0.11	-	-	-
1198	α-terpineol	0.23 ^a^	0.27 ^a^	0.28 ^a^	0.28 ^a^	-	0.29 ^a^	-	-	-
1214	cis-sabinene acetate hydrate	-	-	-	-	-	-	-	-	0.34 ^a^
1235	(*Z*)-3-hexenyl-2-methylbutyrate	0.19 ^a^	0.22 ^b^	0.25 ^c^	-	-	-	-	-	-
1241	cis-3-hexenyl isovalerate	-	-	-	0.26 ^a,d^	0.30 ^b^	0.21 ^c^	0.26 ^a,d^	0.25 ^a^	0.28 ^b,d^
1248	Pulegone	0.26 ^a^	0.42 ^b,d^	0.32 ^c^	0.46 ^b^	0.46 ^b^	0.36 ^c,d^	0.74 ^e^	0.69 ^e^	0.82 ^f^
1261	Piperitone	0.34 ^a^	0.40 ^b^	0.35 ^a^	0.56 ^c^	0.57 ^c^	0.45 ^b^	0.57 ^c^	0.54 ^c^	0.68 ^d^
1334	δ-elemene	-	0.03 ^a^	-	0.05 ^a^	-	-	0.04 ^a^	0.04 ^a^	-
1383	cis-3-hexenyl hexanoate	-	0.05 ^a^	0.06 ^a^	0.08 ^a,b^	0.08 ^a,b^	0.06 ^a^	0.08 ^a,b^	0.07 ^a,b^	0.10 ^b^
1406	(*Z*)-caryophyllene	-	-	0.13 ^a^	-	0.16 ^b^	0.38 ^c^	-	-	0.04 ^d^
1421	(*E*)-caryophyllene	0.47 ^a^	0.7 ^b^	0.45 ^a^	0.79 ^c^	0.19 ^d^	-	0.94 ^e^	0.79 ^c^	1.03 ^f^
1453	α-humulene	-	-	-	-	-	-	0.03 ^a^	-	0.03 ^a^
1481	germacrene D	0.11 ^a^	0.19 ^b,c^	-	0.18 ^b^	-	-	0.24 ^d^	0.19 ^b,c^	0.23 ^d,c^
1497	Bicyclogermacrene	0.04 ^a^	0.07 ^a,b,c^	-	0.08 ^b,c^	-	-	0.09 ^c^	0.08 ^b,c^	0.06 ^a,b^
1575	germacrene d-4-ol	-	-	-	-	-	-	0.04 ^a^	-	-
1584	caryophyllene oxide	-	-	0.06 ^a^	-	0.26 ^b^	-	-	-	0.04 ^c^
1622	Dillapiole	-	-	-	-	-	-	0.05 ^a^	-	-
1630	cis-3-hexenyl phenyl acetate	-	0.04 ^a^	0.04 ^a^	-	0.04 ^a^	-	0.05 ^a,b^	0.04 ^a^	0.07 ^b^
1653	geranyl valerate	-	0.04 ^a^	0.04 ^a^	-	-	-	-	-	-
1654	α-cadinol	-	-	-	0.05 ^a^	0.05 ^a^	-	-	0.05 ^a^	0.05 ^a^
hydrocarbon monoterpenes		-	0.05	0.24	0.54	0.32	0.10	0.37	0.71	0.56
oxygenated monoterpenes		99.38	98.83	98.96	97.98	98.92	99.40	97.92	97.89	97.79
hydrocarbon sesquiterpenes		0.62	1.04	0.7	1.18	0.69	0.44	1.46	1.17	1.53
oxygenated sesquiterpenes		-	0.08	0.08	0.05	0.09	-	0.1	0.09	0.12

RI_C_—calculated from a series of n-alkanes (C8–C40) in a DB-5MS column capillar column; * Mean values followed by the same lowercase letters in the row do not differ at 5% probability.

**Table 3:** ANOVA and Tukey’s test for menthol content in EO.

ANOVA					Tukey’s Test				
Effects	df	SS	MS	*p* *	HT **	HDT **			
						60 min	90 min	120 min	Average HT ***
HT	2	49.4	24.7	<0.001 *	9 h	95.68 ^a^	95.28 ^b^	95.30 ^ab^	95.43 ^A^
HDT	2	0.2	0.1	0.005 *	13 h	93.47 ^d^	94.33 ^e^	94.39 ^e^	94.06 ^B^
Error	23	237,909.3	10,343.9		17 h	92.79 ^f^	92.02 ^g^	91.56 ^h^	92.12 ^C^
Total	27	237,958.9			Average HDT ***	93.98 ^A^	93.88 ^AB^	93.75 ^B^	

Abbreviations: df—degree of freedom; SS—sum of squares; MS—mean of squares; *p*—probability of significance (* significant if *p* ≤ 0.05); HT—harvest time; HDT—hydrodistillation time; ** mean values followed by equal lowercase letters are not differentiated at 5% probability; *** mean values followed by equal upper case letters are not differentiated at 5% probability.

**Table 4:** ANOVA and Tukey’s test for menthone content in EO.

ANOVA					Tukey’s Test				
Effects	df	SS	MS	*p* *	HT **	HDT **			
						60 min	90 min	120 min	Average HT ***
HT	2	11.7	5.8	<0.001 *	9 h	0.72 ^a^	0.6 ^b^	0.84 ^c^	0.72 ^A^
HDT	2	1.3	0.7	<0.001 *	13 h	1.11 ^d^	1.44 ^e^	1.69 ^f^	1.41 ^B^
Error	23	60.32	10,343.9		17 h	1.80 ^g^	2.46 ^h^	2.72 ^i^	2.33 ^C^
Total	27	73.32			Average HDT ***	1.21 ^A^	1.5 ^B^	1.75 ^C^	

Abbreviations: df—degree of freedom; SS—sum of squares; MS—mean of squares; *p*—probability of significance (* significant if *p* ≤ 0.05); HT—harvest time; HDT—hydrodistillation time; ** mean values followed by equal lowercase letters are not differentiated at 5% probability; *** mean values followed by equal upper case letters are not differentiated at 5% probability.

**Table 5:** Phytochemical patterns of *Mentha spicata* L. grown in Brazil.

Location	Major Components	Reference
Espírito Santo (Brazil)	carvone (67.08%), limonene (14.34%), murolene (2.29)	Scherer et al. [39]
Minas Gerais (Brazil)	piperitone (81.18%), piperitenone (14.57%) and limonene (1.47%)	Teixeira et al. [40]
São Paulo (Brazil)	carvone (72.69%), limonene (14.25%) and menthol (2.29%)	Cruz Almeida et al. [41]
Pernambuco (Brazil)	carvone (75.41%), limonene (14.95%) and neomenthol (1.78%)	Braga et al. [42]

**Table 6:** ANOVA and Tukey’s test for EO TAA.

ANOVA					Tukey’s Test				
Effects	df	SS	MS	*p* *	HT **	HDT **			
						60 min	90 min	120 min	Average HT ***
HT	2	772.601	386.300	<0.0001 *	9 h	75.39 ^a^	70.22 ^b^	69.21 ^b^	71.61 ^A^
HDT	2	74.398	37.199	0.0006 *	13 h	69.52 ^b^	66.77 ^b^	68.29 ^b^	68.19 ^B^
Error	23	118,574.414	5155.409		17 h	60.86 ^c^	58.55 ^c^	57.42 ^c^	58.94 ^C^
Total	27	119,421.413			Average HDT ***	68.59 ^A^	65.18 ^B^	64.97 ^B^	

Abbreviations: df—degree of freedom; SS—sum of squares; MS—mean of squares; *p*—probability of significance (* significant if *p* ≤ 0.05); HT—harvest time; HDT—hydrodistillation time; ** mean values followed by equal lowercase letters in the row and column are not differentiated at 5% probability; *** mean values followed by equal upper case letters are not differentiated at 5% probability.

## Data Availability

Data is contained within the article.
